# Association of Dementia With Mortality Among Adults With Down Syndrome Older Than 35 Years

**DOI:** 10.1001/jamaneurol.2018.3616

**Published:** 2018-11-19

**Authors:** Rosalyn Hithersay, Carla M. Startin, Sarah Hamburg, Kin Y. Mok, John Hardy, Elizabeth M. C. Fisher, Victor L. J. Tybulewicz, Dean Nizetic, André Strydom

**Affiliations:** 1 Department of Forensic and Neurodevelopmental Sciences, Institute of Psychiatry, Psychology and Neuroscience, King’s College London, London, England; 2 Division of Psychiatry, University College London, London, England; 3 London Down Syndrome Consortium, London, England; 4 Department of Neurodegenerative Disease, Institute of Neurology, University College London, London, England; 5 Division of Life Science, Hong Kong University of Science and Technology, Hong Kong, Special Administrative Region of China; 6 Reta Lila Weston Institute, Institute of Neurology, University College London, London, England; 7 Department of Neuromuscular Diseases, Institute of Neurology, University College London, London, England; 8 The Francis Crick Institute, London, England; 9 Imperial College, London, England; 10 Lee Kong Chian School of Medicine, Nanyang Technological University, Singapore; 11 Blizard Institute, Barts and the London School of Medicine, Queen Mary University of London, London, England

## Abstract

**Importance:**

This work quantifies the fatal burden of dementia associated with Alzheimer disease in individuals with Down syndrome (DS).

**Objective:**

To explore the association of dementia associated with Alzheimer disease with mortality and examine factors associated with dementia in adults with DS.

**Design, Settings and Participants:**

Prospective longitudinal study in a community setting in England. Data collection began March 29, 2012. Cases were censored on December 13, 2017. The potential sample consisted of all adults 36 years and older from the London Down Syndrome Consortium cohort with 2 data times and dementia status recorded (N = 300); 6 withdrew from study, 28 were lost to follow-up, and 55 had a single data collection point at time of analysis. The final sample consisted of 211 participants, with 503.92 person-years’ follow-up.

**Exposures:**

Dementia status, age, sex, *APOE* genotype, level of intellectual disability, health variables, and living situation.

**Main Outcomes and Measures:**

Crude mortality rates, time to death, and time to dementia diagnosis with proportional hazards of predictors.

**Results:**

Of the 211 participants, 96 were women (45.5%) and 66 (31.3%) had a clinical dementia diagnosis. Twenty-seven participants (11 female; mean age at death, 56.74 years) died during the study period. Seventy percent had dementia. Crude mortality rates for individuals with dementia (1191.85 deaths per 10 000 person-years; 95% CI, 1168.49-1215.21) were 5 times higher than for those without (232.22 deaths per 10 000 person-years; 95% CI, 227.67-236.77). For those with dementia, *APOE *ε4 carriers had a 7-fold increased risk of death (hazard ratio [HR], 6.91; 95% CI, 1.756-27.195). For those without dementia, epilepsy with onset after age 36 years was associated with mortality (HR, 9.66; 95% CI, 1.59-58.56). *APOE* ε4 carriers (HR, 4.91; 95% CI, 2.53-9.56), adults with early-onset epilepsy (HR, 3.61; 95% CI, 1.12-11.60), multiple health comorbidities (HR, 1.956; 95% CI, 1.087-3.519), and those living with family (HR, 2.14; 95% CI, 1.08-4.20) received significantly earlier dementia diagnoses.

**Conclusions and Relevance:**

Dementia was associated with mortality in 70% of older adults with DS. *APOE* ε4 carriers and/or people with multiple comorbid health conditions were at increased risk of dementia and death, highlighting the need for good health care. For those who died without a dementia diagnosis, late-onset epilepsy was the only significant factor associated with death, raising questions about potentially undiagnosed dementia cases in this group.

## Key Points

**Question:**

How does dementia status influence mortality in people with Down syndrome?

**Findings:**

In a longitudinal study including 211 adults with Down syndrome 36 years and older, 27 people died during follow-up (mean, 28; range, 1-65 months), and dementia was the proximate cause of death in 70% of cases. Crude mortality rates were 5 times higher in those with dementia than those without.

**Meaning:**

Nearly all older adults with Down syndrome now have dementia when they die, making this a vital population for researching disease progression, modifying factors, and potential treatments.

## Teaser

This study examines the association of dementia with mortality and examines factors associated with dementia predictors in adults with Down syndrome.

## Introduction

Down syndrome (DS) results from trisomy of chromosome 21 and is associated with multiple health and cognitive comorbidities including congenital heart defects and intellectual disability (ID).^1^ Fifty years ago, life expectancy for those with DS was just 10 years, with congenital heart defects responsible for most deaths within the first year.^2^ Medical advances have since increased mean survival to 63.5 years, yet people with DS still die a mean of 13 years before those without.^3^ Respiratory diseases are now the most frequently cited primary causes of death in adults with DS,^2,3^ with evidence that reduced mobility, poor vision, and epilepsy are each associated with reduced survival in later years.^4^ However, this increasing life expectancy in DS has also revealed an exceptional risk for developing dementia, driven by the near-universal neuropathology of Alzheimer disease (AD) by adulthood.^5^ Understanding how this disease burden is associated with mortality in this aging population is of primary importance for providing appropriate prognostic information, care, and research into potential AD treatments for those both with and without DS.

Alzheimer disease neuropathology in DS stems from triplication of the amyloid precursor protein (*APP*) gene on chromosome 21.^6,7^ Recent mouse model research has found that triplication of other chromosome 21 genes can also increase amyloid-β deposition and worsen cognitive deficits,^8^ although these genes may also have different modulatory and protective roles in AD progression, as suggested by differences between DS-AD and non-DS familial early-onset AD caused by duplication of the *APP* locus alone.^9^ The dementia burden this causes in DS is striking: the mean age of dementia diagnosis is 55 years,^10^ and as many as 88% of this population can be expected to develop dementia by age 65 years.^11^ However, there is great variability in the age of dementia onset, with some individuals surviving past age 60 years with no clear signs of cognitive decline.^12^ Some dementia risk factors seen in the non-DS population similarly play a role in the variability of onset in those with DS. For example, possession of the apolipoprotein E (*APOE*) ε2 allele shows a protective influence, whereas *APOE *ε4 increases dementia risk.^13^ Demographic factors may also influence detection of cognitive decline by caregivers; there is evidence that those who remain living with their family are likely to receive a diagnosis earlier than those in other living situations.^10^

Seizure development is closely linked with dementia in DS. Forty-three percent of those without a previous history of epilepsy develop seizures within a median of 2 years following dementia diagnosis, with most developing generalized tonic-clonic seizures or myoclonic jerks as dementia progresses.^14^ Long-standing epilepsy, present before dementia diagnosis, may shorten survival time after a diagnosis of dementia in individuals with DS,^10^ and there is also evidence that taking antidementia mediation can extend survival.^10^

Although it has been reported that 20 times more people with DS have dementia recorded as a contributory factor on their death certificate than those without DS,^15^ further studies are required to quantify the association that dementia has directly with mortality risk in those with DS, as well as exploring factors that may modify mortality and dementia risk in this population. Better information about factors associated with dementia onset and prognosis will also support the development of clinical trials of treatments.

This study aimed to examine the effect of dementia on crude mortality rates (CMRs) in a large, representative cohort of older individuals with DS in the United Kingdom. Secondary analyses were used to evaluate the influence of additional health and demographic factors on age at death and at dementia diagnosis.

## Methods

### Study Design and Setting

Data were acquired as part of a large, prospective longitudinal study of cognition and health in adults with DS in the United Kingdom.^16^ Ethical approval was secured from the North-West Wales Research Ethics Committee (13/WA/0194). Participation in the primary study was open to all adults with DS, regardless of capacity to consent. Capacity was assessed for each participant at each time, and written informed consent was obtained from all those who were able. A consultee (typically a family member or paid carer) was appointed for individuals without capacity. The consultee was asked to sign a form to indicate their decision about the individual’s inclusion based on their knowledge of the individual and their wishes, in accordance with the UK Mental Capacity Act 2005.

### Participants

Participants were recruited from DS support groups, care homes, existing participant databases, and National Health Services sites in England. Down syndrome status was confirmed genetically where possible (n = 193 of 211 successfully karyotyped). To be eligible for inclusion, participants were required to be 36 years or older at study entry, to have at least 2 data points (mean length of follow-up, 28.66 months; range, 1-65 months). and to have their clinical dementia status known to the informant.

### Data Sources/Measurements

Data for all variables were collected as part of a prospective, longitudinal study. Medical history and demographic details were acquired through a semistructured interview with a carer who knew the participant well. *APOE* genotype was confirmed via blood or saliva sample using a Thermo Fisher Scientific Taqman assay for single-nucleotide polymorphisms rs7412 and rs429358.

### Statistics

Crude mortality rates were calculated using total months of follow-up time for the whole sample and split by dementia status. The Kaplan-Meier method was used to examine survival time for those with and without a dementia diagnosis. To explore factors predicting mortality, Cox proportional hazard models were computed separately for those with and without dementia, using age at exit (or death) as the time variable. Each predictor variable was entered into an independent predictor model in the first instance. Variables significantly associated with mortality were then combined in a final model, using the enter method. To explore factors associated with diagnosis of dementia, Cox regression models were computed using the same predictor variables but using age at diagnosis/exit from study as the timing variable.

### Variables

Time-to-event analyses were computed for death and dementia diagnosis. Dementia status was obtained through carer report, based on independent clinical diagnosis by participants’ regular clinicians after comprehensive clinical assessment. In the United Kingdom, individuals with DS are typically diagnosed as having dementia after specialist assessment in ID services; these expert clinical diagnoses have been shown to be reliable and valid.^17^ To confirm dementia status, 2 ID psychiatrists independently reviewed dementia symptoms for a sample of individuals blind to original clinical diagnoses using items mapping to *International Statistical Classification of Diseases and Related Health Problems, Tenth Revision* and *Diagnostic and Statistical Manual of Mental Disorders* (Fourth Edition) dementia criteria from the structured interview of the Cambridge Examination of Mental Disorders of Older People With Down Syndrome and Others With Intellectual Disabilities.^18^ The presence of significant cognitive decline owing to dementia was confirmed in 86% of individuals and the remaining 14% showing some degree of cognitive decline (possible dementia) (total n = 36). Because dementia is a progressive disease with a prodromal period spanning many years, individuals diagnosed during follow-up were included in the dementia group. Time variables included length in months from study entry to exit for CMR calculations and age in years at event (death or dementia diagnosis) or exit for hazard ratio calculations. The latest data collection point was used as the exit date for censored cases. Cases were censored on December 13, 2017.

Predementia level of ID was obtained via carer report of the participants’ peak level of functioning, based on *International Statistical Classification of Diseases and Related Health Problems, Tenth Revision* characteristics of mild, moderate, severe, or profound ID. Categories were collapsed to produce a binary variable (mild/moderate vs severe/profound).

For the *APOE* variable, participants were grouped for analysis such that those with 2 ε3 alleles formed the reference group, those with 1 or 2 ε2 alleles formed a second group, and those with 1 or 2 ε4 alleles formed the third group. Participants with ε2:ε4 genotype (n = 5) were excluded from *APOE* analyses owing to the opposing effects of alleles ε2 and ε4.

Living situation split those living with family from those in other living situations including supported accommodation, care homes, and residential homes. Additional factors of interest included sex, presence of early-onset (before age 20 years) and late-onset (older than 36 years) epilepsy, hypothyroidism, congenital heart defects, cataracts, dementia medication, antipsychotic medication, obesity (defined as having a body mass index greater than 30 [calculated as weight in kilograms divided by height in meters squared]), and a binary multimorbidity score (0 = none or 1 comorbid condition; 1 = 2 or more conditions). Health conditions included in this score and the list of drugs counted in the medication variables are listed in the eMethods of the Supplement.

## Results

### Sample

Two hundred eleven people (96 women) were included in the final sample, giving 503.92 person-years of follow-up: 344.50 person-years from those without dementia and 159.42 person-years from those who received a clinical dementia diagnosis (n = 66). The mean (SD) age of dementia diagnosis overall was 51.98 (7.09) years (n = 65, data missing from 1 participant); 50.83 (5.72) years for women; and 53.41 (8.38) years for men. Table 1 displays participant characteristics by dementia status.

**Table 1.** Participant Demographics by Dementia Status

Demographic	No. (%)	
	No Dementia	Dementia
Total No. (%)	145 (68.72)	66 (31.28)
Female	60 (41.4)	36 (54.5)
Level of ID		
Mild/moderate	119 (82.1)	50 (75.8)
Severe/profound	25 (17.2)	10 (15.2)
Missing data	1 (0.7)	6 (9.1)
Living situation		
Home with family/partner	31 (21.4)	46 (69.7)
Supported living/care home	113 (77.9)	19 (28.8)
Missing	1 (0.7)	1 (1.5)
Age at entry, mean (SD) [range], y	47.84 (7.29) [36-72]	53.62 (6.94) [38-67]
Age at exit, mean (SD) [range], y	50.23 (7.30) [38-74]	56.05 (7.00) [40-70]
Length of follow-up, mean (SD) [range] mo	28.51 (10.65) [3.0-65.0]	28.98 (12.65) [1.0-55.0]
BMI, mean (SD) [range]	30.16 (6.99) [17.78-56.80 ]	30.79 (7.01) [20.40-54.00]
Obesity (BMI >30)	56 (47.06)	25 (53.19)
Missing data, No.	26	19
Late-onset epilepsy	7 (4.8)	19 (28.8)
Receiving antiepilepsy medication	5 (71.42)	14 (73.68)
Missing data, No.	1	2
Early-onset epilepsy	4 (2.8)	4 (6.1)
Receiving antiepilepsy medication	4 (100)	4 (100)
Receiving antipsychotics (all atypical)	15 (10.34)	10 (15.15)
Hypothyroidism	63 (43.4)	26 (39.4)
Cataracts	32 (22.1)	27 (40.9)
Congenital heart condition	30 (20.7)	7 (10.6)
*APOE* genotype		
ε2:ε2 or ε2:ε3	21 (14.5)	8 (12.1)
ε3:ε3	89 (61.4)	31 (47.0)
ε3:ε4 or ε4:ε4	25 (17.3)	20 (30.3)
ε2:ε4	3 (2.1)	2 (3.0)
Missing data	7 (4.8)	5 (7.6)
≥2 comorbid health conditions	76 (52.4)	34 (51.5)
Receiving antidementia medication	NA	33 (50.0)

Abbreviations: *APOE*, apolipoprotein E; BMI, body mass index (calculated as weight in kilograms divided by height in meters squared); NA, not applicable.

### Crude Mortality Rates

**Figure 1.**
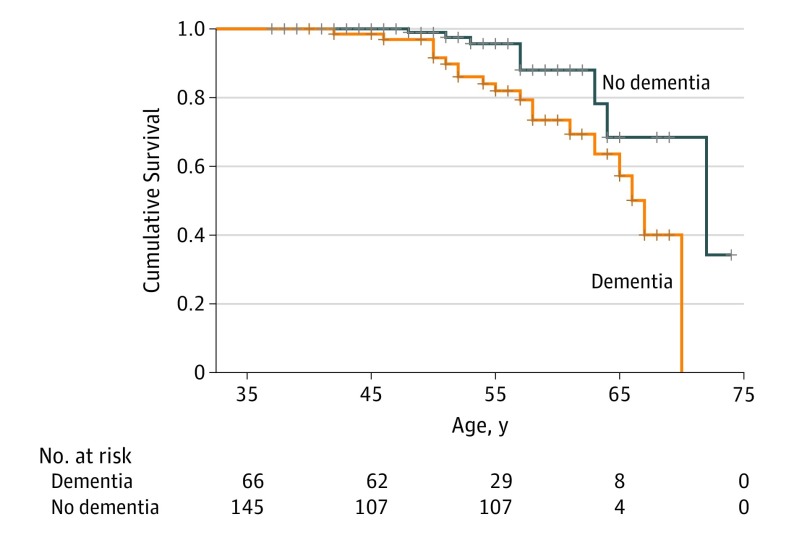
Cumulative Survival by Dementia Status Kaplan-Meier survival curve for individuals with Down syndrome with dementia (n=66) and without dementia (n=145).

Figure 1 shows the Kaplan-Meier survival function for those with and without a diagnosis of dementia; estimated median survival times were 67 and 72 years, respectively. Twenty-seven participants (11 women) died during the follow-up period. The median age at death was 57 years (57 years for men and 54 years for women). Nineteen participants (70.37%) had a clinical diagnosis of dementia, 9 of whom were women (47.37%). Ten men (62.5%) and 9 women (81.8%) who died had a diagnosis of dementia. The median age at death was 57 years for those without dementia and 55 years for those with dementia. During the follow-up period, 28.78% of the dementia group (n = 19) died compared with 5.52% of those without dementia (n = 8). The CMR across the whole sample was 535.80 deaths per 10 000 person-years (95% CI, 529.30-550.30); for those with dementia, the CMR was 1191.85 deaths per 10 000 person-years (95% CI, 1168.49-1215.21); and for those without a dementia diagnosis, the CMR was 232.22 deaths per 10 000 person-years (95% CI, 227.67-236.77).

Of the 8 participants who died without a clinical diagnosis of dementia, 2 had late-onset epilepsy, and 1 was reported to be showing early signs of cognitive decline. One died at aged 50 years of a possible underlying heart condition; 2 died of respiratory diseases with no signs of decline at age 63 years and 73 years, respectively; and for 2 participants the cause of death was unknown.

### Factors Associated With Mortality by Dementia Status

Table 2 shows the Cox regression results for each independent factor split by dementia status and the final combined model for those with dementia. For individuals without a dementia diagnosis, individual Cox regressions revealed that late-onset epilepsy was the only variable associated with mortality, with a near 10-fold increase in risk. For those with a clinical diagnosis of dementia, *APOE* genotype, multimorbidity, early-onset epilepsy, and dementia medication status were all significantly independently associated with mortality, such that presence of 1 or more *APOE *ε4 alleles, 2 or more health conditions, or early-onset epilepsy each were associated with increased mortality risk, and taking antidementia medication was associated with decreased risk. When entered into a combined model, including all significant factors, *APOE* genotype was the only factor to maintain an association at the *P* < .05 level. The presence of at least 1 *APOE *ε4 allele was associated with increased mortality risk nearly 7-fold compared with those with 2 *APOE *ε3 alleles. In our sample, sex was not statistically significantly associated with mortality for those with or without dementia.

**Table 2.** Model Coefficients of Factors Associated With Mortality in Adults With DS

Variable	β Coefficient (SE)	*df*	*P* Value	Hazard Ratio (95% CI)
Adults with DS without dementia				
Independent factors				
Sex	0.8 (0.820)	1	.33	2.23 (0.45-1.11)
Level of ID	−3.28 (5.245)	1	.53	0.04 (0-1102.73)
Multimorbidity status	1.12 (0.767)	1	.14	3.06 (0.68-13.77)
* APOE* genotype		2	.69	
*APOE* group 2 vs group 3	−0.94 (1.097)	1	.39	0.39 (0.045-3.35)
*APOE* group 4 vs group 3	−12.49 (862.96)	1	.99	0
Early-onset epilepsy	−3.02 (44.05)	1	.95	0.049 (0-1.54 × 10^36^)
Late-onset epilepsy	2.27 (0.920)	1	.01	9.66 (1.59-58.56)
Congenital heart defects	0.29 (0.856)	1	.73	1.34 (0.25-7.17)
Antipsychotic medication	−0.07 (1.085)	1	.95	0.93 (0.11-7.82)
Obesity (BMI >30)	0.031 (0.925)	1	.97	1.03 (0.17-6.32)
Hypothyroidism	0.61 (0.721)	1	.40	1.84 (0.45-7.56)
Cataracts	0.06 (0.740)	1	.94	1.06 (0.25-4.52)
Adults with DS and dementia				
Independent factors				
Sex	−0.32 (0.495)	1	.52	0.73 (0.28-1.91)
Level of ID	−0.12 (0.772)	1	.89	0.90 (0.20-4.08)
Multimorbidity status	1.24 (0.530)	1	.02	(1.23-9.80)
*APOE* genotype		2	.01	
*APOE* group 2 vs group 3	−0.28 (0.809)	1	.73	0.75 (0.15-3.68)
*APOE* group 4 vs group 3	1.75 (0.637)	1	.006	5.74 (1.65-19.99)
Early-onset epilepsy	1.87 (0.805)	1	.02	6.50 (1.34-31.47)
Late-onset epilepsy	0.59 (0.490)	1	.23	1.80 (0.69-4.69)
Congenital heart defects	−0.45 (1.036)	1	.67	0.64 (0.08-4.87)
Antipsychotic medication	0.532 (0.586)	1	.36	1.70 (0.54-5.37)
Obesity (BMI >30)	−1.078 (0.692)	1	.12	0.34 (0.09-1.32)
Hypothyroidism	−0.06 (0.506)	1	.90	0.94 (0.35-2.53)
Cataracts	0.44 (0.484)	1	.36	1.55 (0.60-4.01)
Dementia medication status	−1.47 (0.560)	1	.009	0.23 (0.08-0.69)
Final model				
Multimorbidity status	1.27 (0.704)	1	.07	3.57 (0.90-14.20)
* APOE* genotype		2	.009	
*APOE* group 2 vs 3	−0.73 (0.857)	1	.39	0.48 (0.09-2.58)
*APOE* group 4 vs 3	1.93 (0.699)	1	.006	6.91 (1.76-27.20)
Early-onset epilepsy	1.57 (0.896)	1	.08	4.79(0.83-27.69)
Dementia medication status	−0.97 (0.689	1	.16	0.38 (0.10-1.46)

Abbreviations: *APOE*, apolipoprotein E; BMI, body mass index (calculated as weight in kilograms divided by height in meters squared); ID, intellectual disability.

### Factors Associated With Age at Dementia Diagnosis

**Figure 2.**
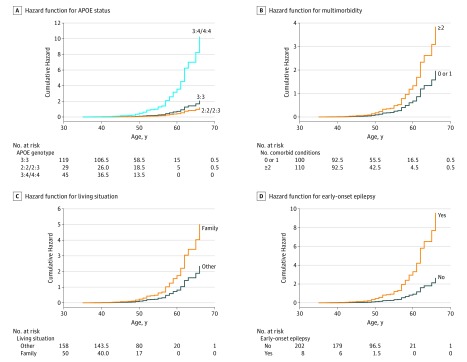
Proportional Hazards of Dementia Predictors Hazard functions for variables associated with dementia diagnosis in Down syndrome.

Sex (women diagnosed earlier), *APOE* genotype, multimorbidity, early-onset epilepsy, and living situation were found to be independently associated with age at dementia diagnosis. All but sex remained significantly associated in the combined model (Table 3, with hazard functions in Figure 2). Increased risk for developing dementia was seen for those carrying at least 1 *APOE *ε4 allele (5-fold increase compared with 2 *APOE *ε3 alleles), having 2 or more comorbid health conditions (2-fold increase), and having early-onset epilepsy (near 4-fold increase). Those living with family were diagnosed at an earlier age. In this sample, carrying an *APOE *ε2 allele was not found to be protective compared with those with 2 *APOE *ε3 alleles (*P* = .36).

**Table 3.** Model Coefficients for Factors Associated With Dementia

Variable	β Coefficient (SE)	*df*	*P* Value	Hazard Ratio (95% CI)
Independent factors associated with dementia				
Sex (women vs men)	−0.581 (0.253)	1	.02	0.56 (0.34-0.92)
Level of ID	0.265 (0.352)	1	.45	1.30 (0.65-2.60)
Early-onset epilepsy	1.716 (0.532)	1	.001	5.56 (1.96-15.79)
Multimorbidity status	0.531 (0.254)	1	.04	1.70 (1.03-2.80)
Congenital heart defect	−0.294 (0.402	1	.46	0.75 (0.34-1.64)
*APOE* genotype		2	<.001	
*APOE* group 2 vs group 3	−0.245 (0.4)	1	.54	0.78 (0.36-1.71)
*APOE* group 4 vs group 3	1.56 (0.324)	1	<.001	4.76 (2.52-8.97)
Cataracts	0.339 (0.253)	1	.18	1.40 (0.85-2.30)
Living situation (family vs other)	1.053 (0.29)	1	<.001	2.87 (1.62-5.06)
Antipsychotic medication	0.061 (0.346)	1	.86	1.06 (0.54-2.10)
Obesity (BMI >30)	0.308 (0.298)	1	.30	1.36 (0.76-2.44)
Hypothyroidism	0.133 (0.255)	1	.60	1.14 (0.69-1.88)
Final model				
Sex (women vs men)	0.391 (0.283)	1	.17	1.48 (0.85-2.58)
Living situation (family vs other)	0.758 (0.346)	1	.03	2.14 (1.08-4.20)
Early-onset epilepsy	1.284 (0.596)	1	.03	3.61 (1.12-11.60)
Multimorbidity status	0.671 (0.3)	1	.03	1.96 (1.09-3.52)
*APOE* genotype		2	<.001	
*APOE* group 2 vs group 3	−0.543 (0.433)	1	.21	0.58 (0.25-1.36)
*APOE* group 4 vs group 3	1.593 (0.339)	1	<.001	4.92 (2.53-9.56)

Abbreviations: *APOE*, apolipoprotein E; BMI, body mass index (calculated as weight in kilograms divided by height in meters squared); ID, intellectual disability.

## Discussion

This study examined the effect of dementia diagnosis on mortality in a representative cohort of adults with DS in England. Dementia was the proximate cause of death in 70% of our sample overall: 10 men (62.5%) and 9 women (81.8%) had dementia when they died. At least 3 of 8 participants who died without a dementia diagnosis showed signs of cognitive decline and/or seizures; thus, this proportion may be even higher.

These results compare strikingly with mortality statistics for England and Wales: dementia of any subtype is mentioned in 17.5% of death certificates for those 65 years and older; and older than 80 years, dementia is the leading cause of death for 14% and 22% of male and female deaths respectively.^19,20^ In our sample, crude mortality was increased 5-fold for those with dementia (CMR with dementia, 1191.85 deaths per 10 000 person-years vs CMR without dementia, 232.22 deaths per 10 000 person-years), giving a similar mortality rate for those with dementia to that reported for AD dementia in the non-DS population (1070 deaths per 10 000 person-years).^21^

In our sample, we found no clear differences in mortality between men and women, matching previous work showing similar ages at death in men and women with DS.^2^ Women were diagnosed as having dementia up to 3 years earlier than men. This pattern has been previously reported in adults with DS in the United Kingdom^10^; however, our findings only held when sex was considered as an independent factor. In the final model, including *APOE* genotype, multimorbidity, living situation and early-onset epilepsy, the influence of sex on dementia diagnosis was lost.

Seizures are a common feature of AD in those with and without DS, occurring in a quarter of patients without DS with AD^22^ and 40% of patients with DS and AD.^14^ However, late-onset epilepsy was also noted in 7 people (4.8%) without a dementia diagnosis in our study, increasing mortality risk 10-fold. For those without dementia, late-onset epilepsy was the only factor associated with mortality. This raises the question of whether seizures can begin in the absence of other features of dementia in individuals with DS or whether these 7 individuals had significant AD pathology and neurological symptoms but had yet to receive a formal dementia diagnosis. While preexisting ID can make it challenging for decline to be identified in this population, detailed clinical assessments have been found to be robust and valid for those with DS,^17^ and a range of sensitive cognitive batteries have been developed within the past 10 years.^16,23,24,25^ Baseline assessments completed in early adulthood can help to serve as each individual’s reference point, allowing decline to be identified on an individual level in this highly variable population.^26^

For people with DS and dementia, carrying at least 1 *APOE *ε4 allele was associated with increased mortality risk 7-fold. These results suggest people with DS may be particularly vulnerable to the effects of *APOE *ε4 because *APOE *ε4 carriers with AD in the non-DS population show little difference to noncarriers with AD in disease progression or mortality.^27,28^ Our data confirm some of the other associations with mortality previously observed, including the deleterious effect of epilepsy and the potentially beneficial effect of currently available medication such as acetyl-choline esterase inhibitors.^29^ However, other associations were not observed: antipsychotics have been found to double mortality risk in people with dementia in the non-DS population,^30^ yet in our sample we did not find a statistically significant association between antipsychotic use and death in those with or without dementia. Similarly, obesity had no discernible association with death or dementia onset in this study. Our data are from a comparatively small group, with a maximum follow-up time of 65 months. Further research using larger samples over longer periods of time would be valuable to clarify whether these reflect genuine differences in risk in the DS population or simply reflect a lack of power for identifying multiple risk factors in this sample.

*APOE *ε4 genotype, early-onset epilepsy, multimorbidity, and living with family were all associated with earlier dementia diagnoses. Previous studies have shown that *APOE* genotype influences dementia risk in DS in much the same way as in the non-DS population. Similarly, epilepsy has been found to increase risk of AD in the general population, with adults with epilepsy younger than 65 years nearly 40 times more likely and those older than 65 years nearly 7 times more likely to be diagnosed as having AD.^31^ Aside from known vascular risk factors, combined health comorbidities may also increase dementia risk in those without DS, suggesting a role for poor general health in dementia risk.^32^ A further explanation could be that increased interaction with health care services for those with multiple health conditions and increased awareness of change for those living with family may make these groups more likely to receive a dementia assessment and subsequent diagnosis rather than increasing risk of dementia per se.

Because multimorbidity was associated with increased dementia risk and mortality in those who received dementia diagnoses, our results also highlight the need for effective recognition and treatment of common health comorbidities in DS. Individuals with ID experience significant health inequalities,^33^ and evidence suggests that incentivizing general practitioners to offer comprehensive ID health checks increases the number of specific health assessments completed and may thus reduce said health inequalities.^34^ Given that several of the comorbidities we included are treatable, such health checks could have longer-term positive effects than have previously been assessed.

### Limitations

Our data were collected as part of a prospective, longitudinal study of adults with DS, providing extensive health information and cognitive assessments for the individuals. While our sample is large for a study of such detail, we acknowledge that the numbers included are relatively small for an epidemiologic study. Health data were collected via informant report, which may be influenced by reporter bias, their memory, and the relationship between the informer and the individual with Down syndrome.

## Conclusions

Our study shows that most adults with DS now have dementia when they die and are affected by some of the same factors associated with dementia (such as *APOE* genotype) as we see in the non-DS population. These findings support the urgent need for clinical trials of treatments to prevent or delay dementia in those with DS. Finally, we hope that our findings can improve clinical care by identifying factors associated with increased risk for dementia and mortality risk in this population, suggesting the potentially beneficial effects of existing medication options and helping clinicians provide prognostic information for their patients with DS.

## Notes

Supplement. **eMethods**.
