# Supplementary material for: Association of Dementia With Mortality Among Adults With Down Syndrome Older Than 35 Years
Source: JAMA Neurol. 2018 Nov 19;76(2):152–60. doi: 10.1001/jamaneurol.2018.3616 (PMC6439956; doi:10.1001/jamaneurol.2018.3616)
Supplement: Supplement. — eMethods. [file jamaneurol-76-152-s001.pdf]

Supplementary Online Content

Hithersay R, Startin CM, Hamburg S, et al. Association of dementia with mortality among adults with Down syndrome older than 35 years. Published online November 19, 2018. *JAMA Neurol.* doi:10.1001/jamaneurol.2018.3616

eMethods.

This supplementary material has been provided by the authors to give readers additional information about their work.

© 2018 Hithersay R et al. *JAMA Neurol.*

Supplementary Material

Medication data was manually checked by a medical professional for each participant from data collected in the LonDownS study. Any medication that may be prescribed for treatment of epilepsy or dementia was included in these variables. Atypical and typical anti-psychotics were counted separately, however in our final sample all participants receiving anti-psychotics were prescribed atypical anti-psychotics.

Medications received by participants in our final group are as follows:

Dementia-related medications: '*cholinesterase inhibitor', 'Donepezil', 'Aricept', 'Rivastigmine', 'Exelon', 'Galantamine', 'Reminyl', 'Memantine', 'Maruxa', 'Nemdatine'

Epilepsy-related medications: Lamotrigine, Sodium Valproate, Levetiracetam, Clonazepam, Phenobarbitone, Phenytoin

Anti-psychotic medications (all atypical): Olanzapine, Risperidone, Aripiprazole, Sulpiride, Quetiapine, Amisulpride

For the binary health co-morbidity variable, diagnosed presence at any time of any of the health conditions listed below counted as one co-morbidity. People with zero or one co-morbid health conditions formed one group, and those with two or more conditions formed the other. Possible co-morbid health conditions: autism, attention deficit hyperactivity disorder, cerebral palsy, schizophrenia, bipolar disorder, depression, anxiety, addiction, Parkinson's disease, stroke, migraine, epilepsy (early onset), epilepsy (late onset), insomnia, narcolepsy, sleep apnoea, congenital heart defect (any form), recurrent pneumonia, coeliac disease, Crohn's disease, rheumatoid arthritis, psoriasis, eczema, gout, hypothyroid, hyperthyroid, type I diabetes, type II diabetes, reflux, cancer: leukaemia, cancer: solid tumour, cataracts, glaucoma, glue ear.

© 2018 Hithersay R et al. *JAMA Neurol*.
